# Pregnancy‐induced transfer of pathogen‐specific T cells from mother to fetus in mice

**DOI:** 10.15252/embr.202356829

**Published:** 2023-08-23

**Authors:** Dennis Yüzen, Christopher Urbschat, Steven Schepanski, Kristin Thiele, Petra C Arck, Hans‐Willi Mittrücker

**Affiliations:** ^1^ Division of Experimental Feto‐Maternal Medicine, Department of Obstetrics and Fetal Medicine University Medical Center Hamburg‐Eppendorf Hamburg Germany; ^2^ Institute of Immunology University Medical Center Hamburg‐Eppendorf Hamburg Germany

**Keywords:** immunological memory, intrauterine transfer, maternal microchimerism, pregnancy, Immunology, Microbiology, Virology & Host Pathogen Interaction

## Abstract

Neonatal health is determined by the transfer of maternal antibodies from the mother to the fetus. Besides antibodies, maternal cells cross the placental barrier and seed into fetal organs. Contrary to maternal antibodies, maternal microchimeric cells (MMc) show a high longevity, as they can persist in the offspring until adulthood. Recent evidence highlights that MMc leukocytes promote neonatal immunity against early‐life infections in mice and humans. As shown in mice, this promotion of immunity was attributable to an improved fetal immune development. Besides this indirect effect, MMc may be pathogen‐specific and thus, directly clear pathogen threats in the offspring postnatally. By using ovalbumin recombinant *Listeria monocytogenes* (LmOVA), we here provide evidence that OVA‐specific T cells are transferred from the mother to the fetus, which is associated with increased activation of T cells and a milder course of postnatal infection in the offspring. Our data highlight that maternally‐derived passive immunity of the neonate is not limited to antibodies, as MMc have the potential to transfer immune memory between generations.

## Graphical abstract

Preconceptual infection of mice with ovalbumin recombinant *Listeria monocytogenes* leads to the formation of OVA‐specific memory T cells. During pregnancy, OVA‐specific T cells are transferred to the fetus where they promote robustness against infection.

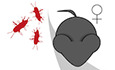

## Introduction

The growing fetus strongly depends on factors provided by the mother. These include nutrients and oxygen, but also hormones, growth factors, and immune mediators. The latter include maternal immunoglobulin (Ig) G antibodies, which are actively transferred across the placenta. In this context, it is long known that pathogen‐specific IgG, mounted by the mother upon natural infection or vaccination, reduce the risk for early‐life infections in the neonate (Jennewein *et al*, 2017; Albrecht *et al*, 2022). More recently, it has been increasingly recognized that cells are also vertically transferred from mother to fetus (Hall *et al*, 1995; Kinder *et al*, 2017). Maternal cells within the fetal organism are rare in number and originate from the genetically disparate mother, hence, they are termed maternal microchimeric cells (MMc). MMc transfer commences upon completion of placentation, the second trimester in humans and around gestation day (gd) 9 in mice (Lo *et al*, 1998; Mold *et al*, 2008). Upon transfer, MMc seed into a variety of fetal organs and can persist in the offspring for years to decades (Piotrowski & Croy, 1996; Lo *et al*, 1998; Maloney *et al*, 1999; Stelzer *et al*, 2015). Phenotypically, MMc mirror the cellular heterogeneity of the maternal organism and can also adopt different cellular phenotypes in the offspring (Vernochet *et al*, 2005; Nijagal *et al*, 2011). Interestingly, a large proposition of MMc are comprised of distinct immune cell subsets (Loubiere *et al*, 2006; Leveque & Khosrotehrani, 2014).

Until today, there is still ongoing debate on the exact functional role of MMc. Several reports indicate a beneficial effect for the development of the offspring's immune system (Mold *et al*, 2008; Kinder *et al*, 2015). In this context, it has recently been shown that MMc can skew hematopoiesis towards the generation of myeloid immune cells (Stelzer *et al*, 2021). As a result, neonates show a less severe course of early‐life infections in mice, whilst human male neonates are more protected from respiratory infections (Stelzer *et al*, 2021). Conversely, several studies linked MMc with disadvantages for offspring's health, such as a higher risk for autoimmunity as demonstrated in patients suffering from diabetes type 1 or in transgenic mouse models of the disease and induction of graft‐versus‐host disease in mice (Neelson *et al*, 2007; Roy *et al*, 2011; Leveque *et al*, 2014).

Given that a large proportion of MMc are leukocytes and considering that the mother has experienced a multitude of infections and likely received an array of vaccinations prior to pregnancy, it can be assumed that these immune MMc also include pathogen‐specific T cells. Hence, the transfer of pathogen‐specific immune cells could provide pathogen‐specific protection of the neonate, which—given the longevity of T cells—could exceed the protection conveyed by maternal IgG (Bianchi *et al*, 2021; Karlmark *et al*, 2021). This is especially relevant during the first year of life, before the recommended vaccination schemes are initiated.

Two case reports underpin this notion, as maternal CD8^+^ T cells specific for human cytomegalovirus (CMV) or Epstein–Barr virus (EBV) could be identified in two 3‐months old patients both suffering from severe combined immunodeficiency. *In vitro* analysis revealed that these cells exerted antiviral function by producing inflammatory cytokines in response to CMV or EBV antigens, respectively (Touzot *et al*, 2012; Koh *et al*, 2021).

Regarding the recent progress in understanding possible pathogen‐specific functions of MMc, we here aimed to illuminate the role of MMc in bacterial infections in a mouse model. Our findings show that preconceptual infection with ovalbumin recombinant *Listeria monocytogenes* (LmOVA) leads to an enrichment of T cells in the uterus accompanied by the seeding of OVA‐specific T cells into the uterus and secondary lymphatic organs. We further provide evidence that OVA‐specific T cells are transferred to the fetus. The presence of these OVA‐specific MMc correlates with less weight loss following early‐life infections.

## Results and Discussion

### LmOVA infection prior to pregnancy induces the accumulation of CD4 ^+^ and CD8 ^+^ T cells in the uterus

**Figure 1:**
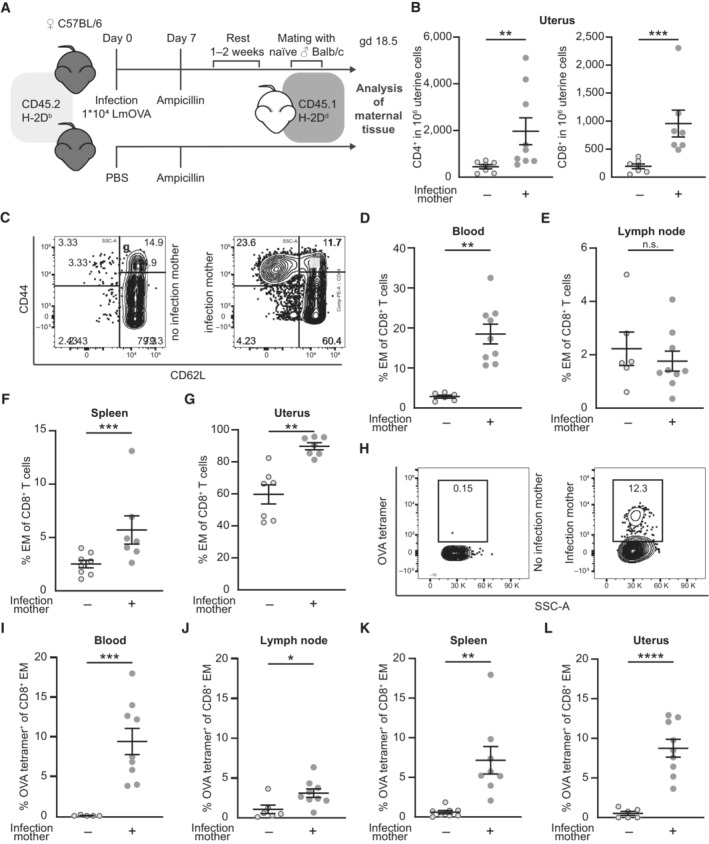
LmOVA infection prior to pregnancy induces the accumulation of CD4^+^ and CD8^+^ T cells in the uterus AExperimental approach.BNumbers of CD4^+^ and CD8^+^ in 1 × 10^6^ uterine cells on gd 18.5 after preconceptual infection of the mother in comparison with non‐infected mothers (*n* = 7, *n* = 9, *n*: biological replicates). T cells were identified as CD45^+^CD3^+^ and either CD4^+^ or CD8^+^ cells.CRepresentative dot plots of CD8^+^ T cells from peripheral blood divided in CD44^+^CD62L^−^ effector/effector memory (EM), CD44^+^CD62L^+^ central memory (CM), and CD44^−^CD62L^+^ naïve subpopulations. Left: non‐infected pregnant control mouse, right: previously infected pregnant mouse.D–GPercentage of EM T cells (CD44^+^, CD62L^−^) among CD8^+^ T cells; (D) peripheral blood (*n* = 6, *n* = 9), (E) uterus‐draining lymph nodes (*n* = 6, *n* = 9), (F) spleen (*n* = 7, *n* = 8), (G) uterus (*n* = 7 each); *n*: biological replicates.HRepresentative dot plots of ovalbumin‐specific CD8^+^ EM T cells stained with H‐2K^b^ ovalbumin_257‐264_ tetramers (OVA tetramers) from peripheral blood. Left: non‐infected pregnant control mouse, right: previously infected pregnant mouse.I–LPercentage of ovalbumin‐specific CD8^+^ EM T cells; (I) peripheral blood (*n* = 7, *n* = 9), (J) uterus‐draining lymph nodes (*n* = 7, *n* = 9), (K) spleen (*n* = 8 each), (L) uterus (*n* = 6, *n* = 9); *n*: biological replicates. Data information: In (B), (D–G), (I–L), data are presented as mean ± SEM. *P ≤ 0.05; **P ≤ 0.01; ***P ≤ 0.001; ****P ≤ 0.0001 (E, J, K, L: Student's *t*‐test; B, D, F, G, I: Mann–Whitney‐*U* test). Source data are available online for this figure.

**Figure EV1:**
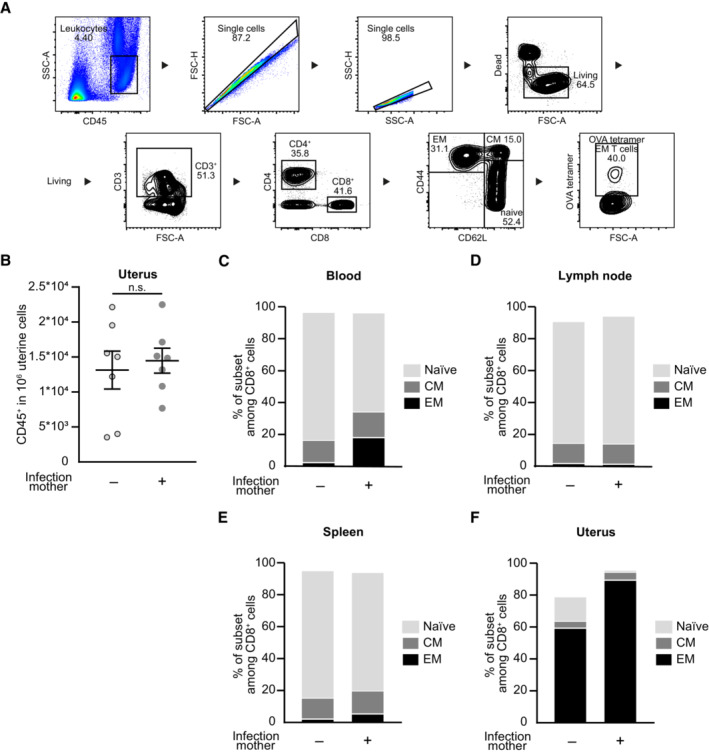
Composition of CD8^+^ T cell subpopulations after preconceptual infection AGating strategy of CD8^+^ T effector/effector memory cells. Blood of pregnant mice at gd 18.5 after preconceptual infection.BNumber of CD45^+^ cells in 1 × 10^6^ uterine cells on gd 18.5 after preconceptual infection of mothers in comparison with naïve mothers (*n* = 7 each, *n*: biological replicates).C–FPercentage of CD8^+^ T cell subpopulations: CD44^+^CD62L^−^ effector/effector memory (EM), CD44^+^CD62L^+^ central memory (CM), CD44^−^CD62L^+^ naïve; (C) peripheral blood (*n* = 6, *n* = 9), (D) uterus‐draining lymph nodes (*n* = 6, *n* = 9), (E) spleen (*n* = 8, *n* = 7), (F) uterus (*n* = 7 each); *n*: biological replicates. Data information: In (B), data are presented as mean ± SEM. In (C–F), data are presented as mean (B, D: Student's *t*‐test; C, E, F: Mann–Whitney‐*U* test).

Infection of mice with LmOVA results in a T cell response to *L. monocytogenes* including a strong CD8^+^ T cell response to the immunodominant peptide ovalbumin_257‐264_ (SIINFEKL), followed by the formation of specific memory T cells. We here infected C57BL/6 female adult mice with LmOVA prior to pregnancy (Fig 1A). In order to ensure complete clearance of LmOVA, we treated mice with ampicillin 7 days after infection. Females were then allogenically mated to Balb/c males. On gestation day (gd) 18.5 and approx. 5 weeks post infection with LmOVA, we detected a significant increase of CD4^+^ and CD8^+^ T cells in the uterus of mice with preconceptual infection compared to non‐infected females (Fig 1B, Gating Strategy Fig EV1A), whereas overall CD45^+^ leukocyte numbers did not significantly differ in the uteri of previously infected and control mice (Fig EV1B). Our observation regarding the uterus is consistent with the general ability of activated T cells to traffic to nonlymphoid tissues (Masopust *et al*, 2001, 2004; Beura *et al*, 2019), which include the different compartments of the female reproductive tract, for example, the ovary, vagina, cervix, and uterus (Suvas *et al*, 2007; Nakanishi *et al*, 2009; Steinert *et al*, 2015).

We next characterized the phenotype of CD8^+^ T cells in uterus, blood, uterus‐draining lymph nodes and spleen of these mice. Naïve T cells were defined as CD62L^+^CD44^−^, central memory (CM) T cells as CD62L^+^CD44^+^ and effector/effector memory (EM) T cells as CD44^+^CD62L^−^ (Fig 1C). We detected higher frequencies of EM T cells within the CD8^+^ T cell subset in peripheral blood and spleen of previously infected females, whilst frequencies were similar to non‐infected females in the uterus‐draining lymph nodes (Figs 1D–F and EV1C–E). The majority of CD8^+^ T cells in the uterus was comprised of EM T cells and this subset was further enlarged after infection with LmOVA (Figs 1G and EV1F). We then assessed the percentage of ovalbumin‐specific T cells within the CD8^+^ EM T cells, using H‐2K^b^ ovalbumin_257‐264_ tetramers (OVA tetramers) (Fig 1H). Here, we could confirm that after LmOVA infection up to 10% of CD8^+^ EM T cells in blood, uterus‐draining lymph nodes, spleen, and uterus were ovalbumin‐specific (Fig 1I–L). Similar accumulation of memory T cells in the female reproductive tract has been demonstrated in mouse and humans after viral infection, for example, after infection with HIV‐1, HSV‐2, CMV and EBV (Zhu *et al*, 2009; Tang & Rosenthal, 2010; van der Zwan *et al*, 2018), or bacterial infection with chlamydia (Nguyen *et al*, 2020; Labuda *et al*, 2021). Given the fact that the overall number of CD4^+^ and CD8^+^ T cells and the percentage of EM CD8^+^ T cells remain elevated even after weeks following initial infection, they may have formed a tissue‐resident T cell subset (Davé *et al*, 2021; Peng *et al*, 2021). Screening for additional cellular markers such as CD69, an established marker for tissue‐residency, or CD103, a typical marker for resident T cells within mucosal tissues, would be needed to validated this hypothesis (Topham & Reilly, 2018). Further, laboratory mice reared in conventional animal facilities, an environment controlled for pathogens and commensals, show immunologically naïve peripheral organs (Beura *et al*, 2016; Abolins *et al*, 2017). The immunologically naïve state of the uterine tissue in laboratory mice raises questions regarding the effect of preconceptual or perinatal infection models in pregnancy research with limited external validity for adult women. This underlines the need for comparable analyses in wild‐derived mouse models, as postulated by different research groups (Beura *et al*, 2016; Huggins *et al*, 2019; Rosshart *et al*, 2019).

### Intergenerational transfer of OVA‐specific MMc from mother to offspring

**Figure 2:**
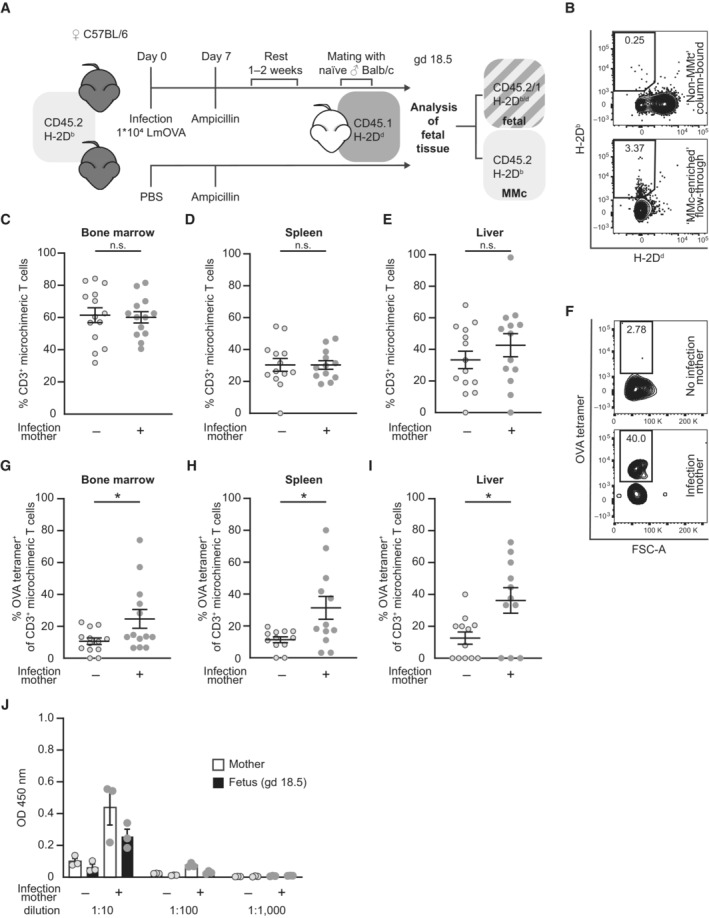
Intergenerational transfer of OVA‐specific MMc from mother to offspring AInfection and mating strategy and identification strategy for maternal microchimeric cells (MMc) in offspring of CD45.2^+^ H‐2D^b+^ C57BL/6 females mated with CD45.1^+^ H‐2D^d+^ Balb/c males.BRepresentative dot plots of column bound ‘non‐MMc’ fraction (upper) and ‘MMc‐enriched’ flow‐through fraction (lower) after negative selection of MMc via antibody labeling of paternal H‐2D^d^ and magnetic‐activated cell sorting (MACS) gated on CD45.2^+^CD45.1^−^ cells.C–EPercentage of CD3^+^ T cells of total MMc; (C) fetal bone marrow (*n* = 13, *n* = 14), (D) fetal spleen (*n* = 12, *n* = 13), (E) fetal liver (*n* = 13, *n* = 14); *n*: biological replicates.FRepresentative dot plots for OVA‐tetramer staining of CD3^+^ MMc from spleens of fetuses of previously infected or non‐infected mothers.G–IPercentage of OVA‐specific MMc stained with OVA tetramers among CD3^+^ microchimeric T cells; (G) bone marrow (*n* = 13 each), (H) spleen (*n* = 12 each), (I) liver (*n* = 11, *n* = 12); *n*: biological replicates.JSerum anti‐Lm IgG titer of mothers and fetuses at gd 18.5 after preconceptual infection of the mother with LmOVA; *n* = 3 biological replicates, each averaged from *n* = 2 technical replicates). Data information: In (C–E), (G–J), data are presented as mean ± SEM. *P ≤ 0.05 (C, D, E, H: Student's *t*‐test; G, I: Mann–Whitney‐*U*). Source data are available online for this figure.

**Figure EV2:**
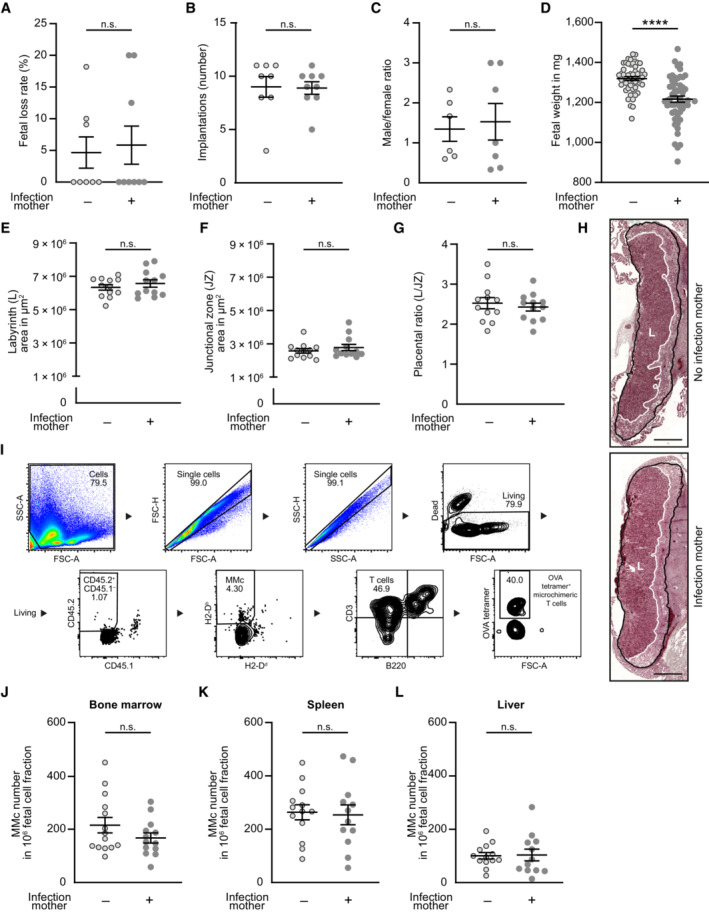
No differences in pregnancy outcome and MMc numbers in fetal organs after preconceptual infection of female mice A–DPregnancy outcome parameter: (B) Percentage of fetal loss rate (*n* = 8, *n* = 9); (C) Number of implantations on gd 18.5 (*n* = 7, *n* = 9); (D) Male/female ratio of offspring (*n* = 6, *n* = 7); (E) Fetal weight on gd 18.5 (*n* = 54, *n* = 55); *n*: biological replicates.E–GPlacental evaluation: (E) Area of placental labyrinth (L) (*n* = 12 each); (F) Area of placental junctional zone (JZ) (*n* = 12–13); (G) Ratio of placental labyrinth to the junctional zone (L/JZ ratio) as an indicator for placental function on gd 18.5 (*n* = 12 each); *n*: biological replicates.HRepresentative depiction of murine placenta assessment, highlighted for labyrinth (L, white) and labyrinth + junctional zone (black). Scale bar = 1,000 μm.IGating strategy of ‘MMc‐enriched’ flow‐through. Bone marrow of fetal mice at gd 18.5 born to a preconceptually infected mother.J–LNumbers of MMc in 1 × 10^6^ fetal cells on gd 18.5; (J) bone marrow (*n* = 14, *n* = 13), (K) spleen (*n* = 14, *n* = 12), (L) liver (*n* = 13, *n* = 12); *n*: biological replicates. Data information: In (B–H), (J–L), data are presented as mean ± SEM. ****P ≤ 0.0001 (C, E, G, K, L: Student's *t*‐test; A, B, D, F, J: Mann–Whitney‐*U* test).

Fetal mice were analyzed at gd 18.5—one day before birth—to exclude possible confounders regarding the offspring's immune response affecting MMc numbers, for example, microbial colonization at birth or maternal breastfeeding. We first analyzed pregnancy outcome parameter and observed similar fetal loss rate, number of implantations and male/female fetal sex ratio between preconceptually infected mothers and non‐infected control mothers (Fig EV2A–C). However, a significantly lower fetal weight was detected on gd 18.5 if the mother had been infected with LmOVA prior to pregnancy (Fig EV2D). Placental dysmorphology after preconceptual infection of the mother could be excluded by placental histology. Histomorphological evaluation of placental tissue showed no differences in size of placental areas and placental ratio (Fig EV2E–H). To identify MMc in the offspring's tissue, we applied an established allogenic mating model, in which MMc are detected based on the expression of CD45 variants and the H‐2D haplotype (Stelzer *et al*, 2021). Leukocytes from female mice were homozygous for CD45.2 and H‐2D^b^, whereas those of males were homozygous for CD45.1 and H‐2D^d^. Consequently, MMc can be detected by selecting for CD45.2^+^CD45.1^−^ and H‐2D^b+^H‐2D^d−^ cells among CD45.1^+^CD45.2^+^ and H‐2D^b+^ H‐2D^b+^ fetal cells (Fig 2A). Due to the sparsity of MMc among fetal cells, they were enriched after tissue‐processing utilizing magnetic‐activated cell sorting. Fetal cells expressing the paternal markers CD45.1 and H‐2D^d^ were labeled with magnetic beads and retained in the column by applying a magnetic field, whereas CD45.1^−^ and H‐2D^d‐^ MMc were enriched in the flow‐through (Fig 2B, Gating Strategy Fig EV2I). MMc numbers in bone marrow, spleen, and liver were not altered in fetuses of preconceptually infected mothers (Fig EV2J–L). Similarly, percentages of microchimeric CD3^+^ T cells were consistent between fetuses of previously infected and non‐infected mothers (Fig 2C–E). MMc in the fetal bone marrow, spleen and liver of fetuses were analyzed for OVA‐specific CD3^+^ T cells (Fig 2F–I). Most likely due to the low numbers of analyzed MMc and their heterogeneous cell composition, OVA tetramer staining resulted in a higher background than that observed in adult mice. However, compared to tissues of fetuses from control mothers, we detected a substantial increase in OVA‐specific CD3^+^ T cells in the fetal bone marrow, spleen and liver of fetuses of preconceptually infected mothers, providing evidence for a transfer of OVA‐specific T cells from the mother to the fetus during pregnancy. Comparable percentages of OVA‐specific T cells were found in all three investigated fetal organs, suggesting non‐organ specific distribution of cells in the fetus. This corresponds to our observation within the maternal organism, in which OVA‐specific CD8^+^ EM T cells can be found in the peripheral blood, lymph node, uterus and spleen.

Our study analyses the frequency of OVA‐specific MMc at the end of the third trimester. Since the transfer of MMc starts with the beginning of the second trimester after successful placentation (Rossant & Cross, 2001; Marleau *et al*, 2003; Vernochet *et al*, 2005), similar experiments conducted earlier in pregnancy might provide insights on the initial distribution of MMc in fetal organs. MMc are comprised of various distinct maternal cell types (Loubiere *et al*, 2006). For now, it remains elusive whether cells are specifically selected for the transfer to the fetus, or whether MMc transfer is a result of placental leakage and cell types are selected by chance (Cartwright & Balarajah, 2005; Dawe *et al*, 2007; Dye *et al*, 2001). It is intriguing to speculate on a distinct selection process of maternal cells, which are transported to the placenta and passed to the fetus to adequately prepare the offspring's immune system. Interestingly, a study investigating re‐infection with LmOVA during pregnancy demonstrated higher proportions of OVA‐specific CD8^+^ T cells in the decidua after preconceptual infection of female mice. The authors suggested, that the feto‐maternal interface exhibits special leakiness for T cells primed by infection (Clark *et al*, 2014), which might benefit the transfer of pathogen‐specific MMc to the fetus.

Our data show high variability in the percentage of OVA‐specific T cells among total MMc ranging from 5 to 80%. This mirrors the variation in total MMc numbers as reported for human cord blood samples. MMc numbers in the cord blood vary drastically between mother–child pairs, with some newborns having high MMc numbers and some having none (Harrington *et al*, 2017; Stelzer *et al*, 2021). The reason for this variation between individuals is unknown. Since MMc need to cross the feto‐maternal barrier formed by the placenta, the status of placental tissue might play a decisive role in mediating cell transfer. Activation of the placental tissue, e.g., during maternal infection or other stressors, involves the upregulation of adhesion proteins relevant for cell trafficking (Kretschmer *et al*, 2020; Umapathy *et al*, 2020). However, in‐depth analysis of placental function during an ongoing pregnancy in humans is highly challenging due to limited accessibility.

### Only marginal production and transfer of maternal anti‐listeria antibodies to the offspring

During the first months of life, neonates are protected from infections by active transfer of antibodies from the mother to the fetus over the placenta or via breast milk after birth (Albrecht *et al*, 2022). To test for the induction and transfer of listeria‐specific antibodies, sera from infected and non‐infected mothers and from fetuses at gd 18.5 were analyzed by ELISA against heat‐killed listeria (Fig 2J). Without infection, sera from mothers and fetuses show very weak reactivity to listeria at low dilution of 1:10, which most likely reflects binding of natural antibodies to conserved bacterial structures. Infection caused a slight increase of reactivity at the 1:10 dilution but there was only background reactivity at higher dilutions. Thus listeria infection caused only marginal antibody responses and consequently limited transfer of these antibodies to the fetus.

Antibodies are considered irrelevant for protection against *L. monocytogenes* (Mackaness, 1962; Portnoy *et al*, 2002; Asano *et al*, 2016). However, a recent study could demonstrate that pregnancy causes altered glycosylation of IgG. After transfer, these antibodies can bind inhibitory receptors on regulatory B cells of the offspring and thereby limit IL‐10 production by these cells. In the case of listeria infection of neonates, maternal listeria‐specific antibodies could override suppression by regulatory B cells resulting in enhanced protection (Erickson *et al*, 2022). In their study, Erickson *et al*. use infection protocols which result in high anti‐listeria IgG titers with strong reactivity at 1:1,000 dilutions in sera of mothers, which strikingly differs from the anti‐listeria IgG titers observed by us. Thus, we consider the protective effects of intergenerational antibody transfer in our study to be of less relevance.

### Absence of innate immune activation after preconceptual infection and re‐infection of pregnant mice

**Figure 3:**
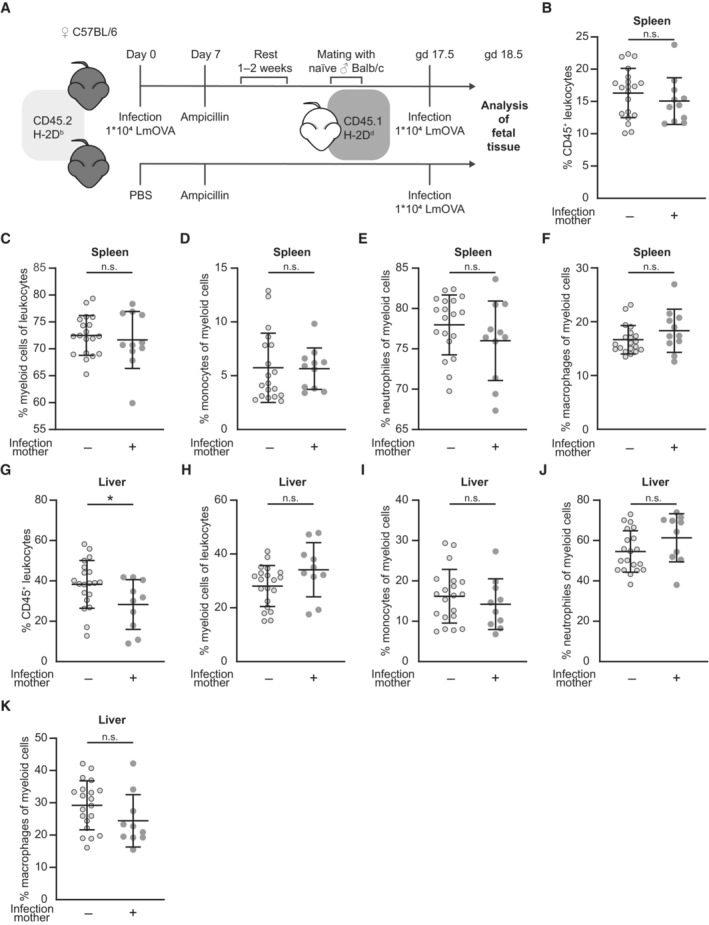
No fetal innate immune activation after preconceptual infection and re‐infection of pregnant mice AExperimental approach.B–FPercentages of innate immune cell subsets in the fetal spleen; (B) CD45^+^ leukocytes (*n* = 20, *n* = 11), (C) CD45^+^CD11b^+^ myeloid cells (*n* = 19, *n* = 11), (D) CD45^+^CD11b^+^Gr‐1^−^ monocytes (*n* = 19, *n* = 11), (E) CD45^+^CD11b^+^Gr‐1^+^ neutrophils (*n* = 19, *n* = 11) and (F) CD45^+^CD11b^+^F4/80^+^ macrophages (*n* = 20, *n* = 11); *n*: biological replicates.G–KPercentages of innate immune cell subsets in the fetal liver; (G) CD45^+^ leukocytes (*n* = 20, *n* = 10), (H) CD45^+^CD11b^+^ myeloid cells (*n* = 20, *n* = 10), (I) CD45^+^CD11b^+^Gr‐1^−^ monocytes (*n* = 20, *n* = 10), (J) CD45^+^CD11b^+^Gr‐1^+^ neutrophils (*n* = 20, *n* = 10) and (K) CD45^+^CD11b^+^F4/80^+^ macrophages (*n* = 20, *n* = 10); *n*: biological replicates. Data information: In (B–K), data are presented as mean ± SEM. *P ≤ 0.05 (B, C, G–I: Ordinary one‐way ANOVA; D‐F, K, J: Kruskal–Wallis test). Source data are available online for this figure.

In order to determine whether infection of the mother caused inflammation in tissues of the fetus, we characterized the myeloid cell compartment of fetal spleen and liver at gd 18.5 after preconceptual infection of the mother. To amplify the maternal immune response, pregnant mothers were re‐infected with LmOVA at gd 17.5 (Fig 3A). In the fetal spleen, we did not detect differences in the frequencies of total leukocytes (Fig 3B) and myeloid cells (Fig 3C). Among myeloid cells, no changes in frequencies were detected for monocytes (Fig 3D), neutrophils (Fig 3E) and macrophages (Fig 3F). Livers of fetuses with an infected mother had even slight lower frequency of total leukocytes (Fig 3G). However, the composition of myeloid cell subsets did not differ from that of controls (Fig 3H–K). Thus, infection of the mother did not cause inflammation with accumulation of myeloid cells in fetal tissue.

### Offspring born to a preconceptually infected mother are less susceptible to infection

**Figure 4:**
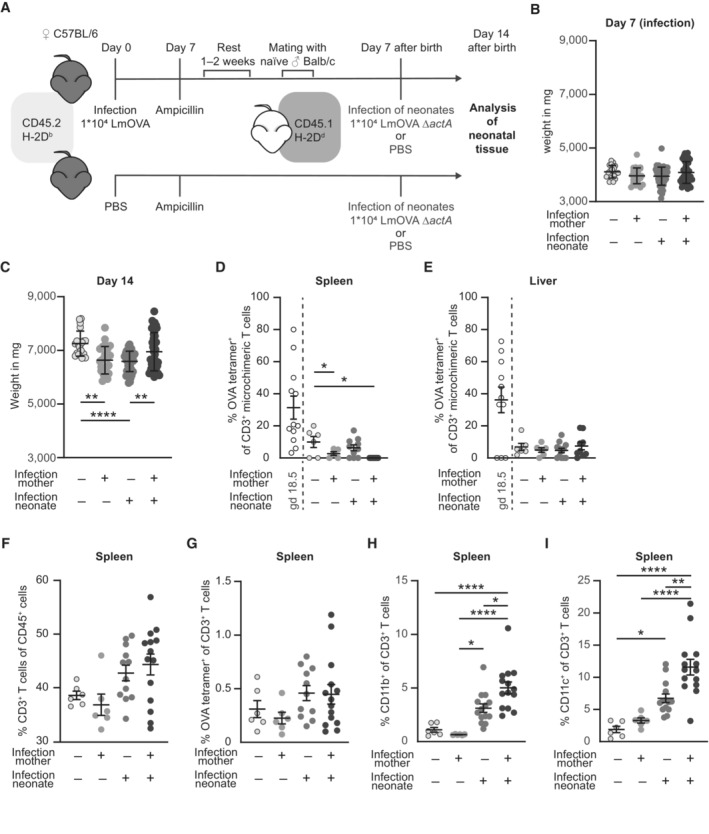
Offspring born to a preconceptually infected mother are less susceptible to infection and show activation of CD3^+^ T cells AExperimental approach.B, CNeonatal body weight in mg (*n* = 20–55). (B) Day 7 after birth (day of infection), (C) day 14 after birth (7 days after infection); *n*: biological replicates.D, EPercentages of OVA tetramer^+^ cells among CD3^+^ MMc (percentages of OVA tetramer^+^ among CD3^+^ MMc at gd 18.5 (Fig 2H and I) are included for direct comparison); (D) spleen (*n* = 5–10), (E) liver (*n* = 5–10); *n*: biological replicates.F–IPercentage of (F) CD3^+^ T cells of CD45^+^ spleen cells (*n* = 6–14), (G) OVA‐specific cells of CD3^+^ T cells from spleen cells (*n* = 6–14), (H) CD11b^+^ cells of CD3^+^ T cells from spleen cells (*n* = 6–14), and (I) CD11c^+^ cells of CD3^+^ T cells from spleen cells (*n* = 6–14); *n*: biological replicates. Data information: In (B–I), data are presented as mean ± SEM. *P ≤ 0.05; **P ≤ 0.01; ****P ≤ 0.0001 (B–D, G: Ordinary one‐way ANOVA; E, F, H, I: Kruskal–Wallis test). Source data are available online for this figure.

**Figure EV3:**
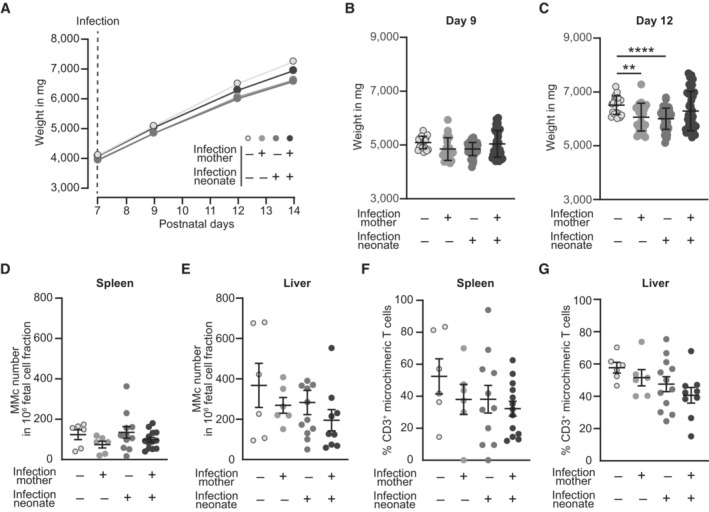
Infection alters neonatal weight but does not influence MMc numbers ABody weight development after infection of neonates depicted as weight in mg.B, CNeonatal body weight in mg (*n* = 20–55, *n*: biological replicates). (B) Day 9 after birth (2 days after infection), (C) day 12 after birth (5 days after infection).D, ENumber of MMc in 1 × 10^6^ fetal cells on day 7 after infection; (D) spleen (*n* = 6–14), (E) liver (*n* = 6–11); *n*: biological replicates.F, GPercentage of CD3^+^ T cells among MMc; (F) spleen (*n* = 6–13), (G) liver (*n* = 6–12); *n*: biological replicates. Data information: In (B–G), data are presented as mean ± SEM. **P ≤ 0.01; ****P ≤ 0.0001 (B, F, G: Ordinary one‐way ANOVA; C–E: Kruskal–Wallis test). Source data are available online for this figure.

After demonstrating the presence of OVA‐specific MMc in gd 18.5 fetuses of preconceptually infected mothers and the absence of inflammation, we analyzed the consequences of preconceptually infection of the mothers on the susceptibility of the offspring to infection. Therefore, neonates were infected with a LmOVA strain deficient for *act*A (LmOVA Δ*act*A) at day 7 after birth (Fig 4A). Due to the deletions of the *act*A gene, the LmOVA Δ*act*A strain is attenuated but still induces a profound immune response without killing the vulnerable neonate (Kollmann *et al*, 2007), which allows to evaluate the response of microchimeric OVA‐specific T cells to infection. As an indicator of overall fitness, neonatal weight was monitored at 4 time points starting with the day of infection (Figs 4B and C and EV3A–C). Assessment of neonatal weight after infection at day 14 after birth—7 days after infection—showed a higher body weight of neonates born to preconceptually infected mothers compared to neonates born to naïve mothers (Fig 4C), indicating a higher robustness of offspring to infection.

### No expansion of OVA‐specific MMc after infection

MMc were still present in the neonatal spleen and liver at day 14 after birth (7 days after Infection) with LmOVA Δ*act*A (Fig EV3D and E). Additionally, microchimeric CD3^+^ T cell numbers were comparable in neonates born to previously infected and control mothers (Fig EV3F and G). However, at this time point we detected only background levels of OVA‐specific T cells among CD3^+^ microchimeric T cells from spleen and liver of neonates born to a preconceptually infected mother when compared to levels at gd 18.5 (Fig 4D and E). Although listeria mainly localize to the spleen and liver due to their uptake by resident phagocytes, infection did not cause expansion of residual OVA‐specific T cell MMc in response to LmOVA Δ*act*A infection of the neonate. This was not due to a failure of LmOVA Δ*act*A to induce a T cell response since we observed expansion of CD3^+^ T cells and formation of OVA‐specific CD3^+^ T cells in spleens of neonatally infected animals (Fig 4F and G). Antigen‐experienced MMc might therefore have a function other than pathogen clearance. In order to control for the quality of the neonatal T cell response, CD3^+^ T cells were analyzed for activation markers. Activation of CD8^+^ T cells following infection is accompanied by the upregulation of surface proteins, including CD11b and CD11c (Lin *et al*, 2003; Chen *et al*, 2013), which belong to the β2‐integrin family and function as adhesion molecules for various ligands involved in cellular adhesion and phagocytosis (McFarland *et al*, 1992; Huleatt & Lefrançois, 1995). We identified a significantly higher frequency of CD3^+^CD11b^+^ and CD3^+^CD11c^+^ cells in the spleen of infected neonates born to preconceptually infected mothers compared to infected neonates born to control mothers (Fig 4H and I). In conclusion, pathogen‐specific MMc T cells show no expansion after neonatal infection but are associated with an increased activation of fetal CD3^+^ T cells following listeria infection.

Our study is based on a well‐established model using LmOVA to generate OVA‐specific T cells that can be identified using OVA‐tetramers. It would be interesting to validate our findings for pathogen‐specific MMc by using other murine infection models. Further, our experimental approach does not address the transfer of MMc via breast milk (Moles *et al*, 2018). However, the absence of OVA‐specific MMc at day 14 after birth suggests that their transfer occurs intrauterine. Still, this needs to be validated by cross‐fostering experiments. Our findings raise question for the consequences of preconceptual or prenatal infection in women and the possible transfer of pathogen‐specific MMc in the womb. If confirmed, for example, in human cord blood samples, this might add another aspect to our understanding of vaccination strategies for pregnant women.

The transfer of pathogen‐specific MMc broadens our understanding on the transfer of immunological memory. Lining up with the well‐known transport of antibodies over the placenta, MMc add a cellular component to further complete the picture of maternal passive immunity.

## Materials and Methods

### Mice

In accordance with the German Animal Welfare Act and institutional guidelines of the University Medical Center Hamburg‐Eppendorf, animal care and experimental procedures were performed based on the mandatory requirements. The ethical approvals (approval numbers N125/19, G010/17) were issued by the State Authority of Hamburg. C57BL/6J (CD45.2, H‐2D^b^) and Balb/c CD45.1 (CD45.1, H‐2D^d^, CByJ.SJL(B6)‐Ptprca/J) were purchased from The Jackson Laboratory (Bar Harbor, ME). Female mice were housed in groups, whereas male mice were situated separately in the animal facility of the University Medical Center Hamburg‐Eppendorf. Adequate housing was ensured at a room temperature of 21°C and humidity at 43% in a 12‐h light/12‐h dark cycle. Mice were provided with chow and water ad libitum. For experiments female mice were used at 8–10 weeks of age and male mice were used starting from fertile age up until 1 year of age.

### *Listeria monocytogenes* infection

Female mice aged 8–10 weeks were infected with 1 × 10^4^ colony forming units (CFU) of ovalbumin recombinant *L. monocytogenes* (LmOVA) in 100 μl sterile PBS (Thermo Fisher Scientific, Waltham, MA) via the lateral tail vein. Control mice were injected accordingly with 100 μl sterile PBS. One week after injection, both groups were treated with ampicillin (Ratiopharm, Ulm, Germany) in the drinking water (1 g/l) for 7 days and mice were given an additional week of regeneration. For the evaluation of non‐specific fetal immune activation, we re‐infected preconceptually infected female mice during pregnancy at gd 17.5 with 1 × 10^4^ CFU of LmOVA. For neonatal infection at day 7 after being born, we used 1 × 10^4^ CFU of the attenuated ovalbumin recombinant *L. monocytogenes* strain deleted for *act*A (LmOVA Δ*act*A) in 100 μl sterile PBS injected i.p. Control neonates were injected with 100 μl sterile PBS. Concentration of inocula used for infection experiments was verified by plating serial dilutions on TSB agar plates.

### Pregnancy mouse experiment design

To exclude environmental factors on housing and pregnancy maintenance, non‐litter mates of female mice of all groups were kept and mated in the same animal facility. All experiments were repeated with at least three biological replicates of individual fetuses derived from at least three separate litters. Mating of 12–13 week old females with one male was started at 3–5 p.m. for a maximum of 5 consecutive days. Overnight insemination was verified by the presence of a vaginal plug at 8–10 a.m. at the subsequent day and defined as gd 0.5. Body weight measurement at gd 10.5 was used to confirm successful pregnancy (10–15% body weight increase relative to gd 0.5). Fetal loss rate was calculated as the percentage of abortions among implantation sites.

### Tissue‐collection and processing

Fetal tissue was collected by decapitating fetuses on gd 18.5. To sustain homogeneity in fetal size, offspring were exclusively used from mothers giving birth to at least 8 up to 11 neonates. Pregnancies and tissue harvesting were stringently timed to minimize differences in fetal age. Collected fetal tissues (bone marrow from femur and tibia, spleen, liver) and maternal tissues (uterus, liver, spleen and uterus‐draining lymph nodes) were mechanically minced and washed through a filter to obtain single‐cell suspensions. Fetal bone marrow samples were pooled to guarantee adequate cell numbers for subsequent analysis. A similar approach was chosen for fetal spleen samples. For maternal spleen samples, erythrocyte lysis was required. Maternal uterus samples were digested with Accutase^®^ (Stemcell Technologies, Vancouver, Canada). Maternal blood was collected by retroorbital puncture in an EDTA‐coated microvette (Sarstedt, Nümbrecht, Germany). Liver and spleen of neonates were selected for further analysis due to being the primary infection site of *L. monocytogenes* infection. Neonatal tissues (liver and spleen) were collected at day 14 after birth by decapitating neonatal mice and processed as described. Neonatal spleen samples were pooled to guarantee adequate cell numbers. Additionally, neonatal liver cells were purified using Percoll (GE Healthcare, Chicago, IL) gradient.

### Enrichment of MMc by magnetic‐activated cell sorting

To allow reliable characterization of MMc, MMc within the bone‐marrow, spleen and liver sample were enriched based on their lack of H‐2D^d^ expression by magnetic‐activated cell sorting (MACS) following published protocols for low frequency cell subsets (Moon *et al*, 2007). In detail, two fetal spleens of litter mates were pooled to ensure a sufficient amount of fetal cells. Similarly, cells from fetal hind and front leg bones originated from two litter mates were pooled. Fetal livers were individually processed. Harvested cells were incubated for 30 min at 4°C with normal rat serum and anti‐mouse CD16/32 (Biolegend, San Diego, CA) in order to block unspecific FcγRII/III binding. To separate fetal from maternal cells, fetal cells were stained with APC‐conjugated anti‐H‐2D^d^ antibody for 30 min at 4°C in the dark. Subsequent incubation with magnetically labeled anti‐APC MicroBeads (Miltenyi Biotec, Bergisch Gladbach, Germany) for 15 min at 4°C, enabled separation through MACS columns (Miltenyi Biotec, Bergisch Gladbach, Germany). While unlabeled MMc (H2‐D^d^ negative and H2‐D^b^ positive) were passing the column and were collected in the flow‐through fraction, fetal cells (H2‐D^d^ positive and H2‐D^b^ negative) were retained within the column and later eluted.

### Flow cytometry

Cells were incubated with normal rat serum and anti‐mouse CD16/32 in order to block unspecific FcγRII/III binding. Fluorochrome‐conjugated antibodies, staining for cell viability and H‐2K^b^ chicken ovalbumin amino acid 257–264 (SIINFEKL) tetramers conjugated to Brilliant Violet 421 (provided by the National Institutes of Health [NIH] Tetramer Core Facility, Atlanta, GA) were added to the cell suspension and incubated for 30 min at 4°C in the dark. Fluorochrome conjugated mAb against CD3ε (clone 145‐2C11), B220 (RA3‐6B2), CD4 (RM4‐5), CD44 (PE), CD45 (30‐F11) CD62L (MEL‐14), CD8 (53‐6.7), CD11b (M1/70), CD11c (N418), CD45.1 (A20), CD45.2 (104), H2‐D^b^ (KH95), H2‐D^d^ (34‐1‐2S), Dead/Live were purchased from BD Pharmingen (Franklin Lakes, NJ), eBiosciences (Waltham, MA), or Biolegend. To identify MMc among fetal cells, CD45 and MHC class I expression was evaluated. Maternal origin of cells was verified by CD45.2 expression by concurrent absence of CD45.1. Subsequent determination of MHC class I marker H‐2D^b^ expression and H‐2D^d^ absence was used to refine the gating strategy. After identification, MMc were further characterized based on specific lineage marker. Samples were acquired using a A3 flow cytometer (Becton Dickinson, Franklin Lakes, NJ) using 1 × 10^6^ cells per fetal bone marrow, fetal liver and neonatal samples and 0.2 × 10^6^ cells for fetal spleen if possible. Absolute MMc number was calculated depending on their relative abundance among fetal cells. Doublets and dead cells were excluded in the gating process. Data analysis was performed using FlowJo software v10 (Becton Dickinson, Franklin Lakes, NJ). To control for adequate gating of cell populations, fluorescence minus one (FMO) controls containing all antibodies of a staining panel except one were used.

### ELISA

Listeria‐specific antibodies in sera from adult and fetal mice at gd 18.5 after preconceptual infection were assessed with enzyme‐linked immunosorbent assay (ELISA). Plates were coated overnight with heat‐killed *L. monocytogenes* and subsequently blocked with PBS 1% BSA. Sera were incubated in duplicates in 1:10, 1:100 and 1:1,000 dilutions followed by incubation with secondary anti‐mouse IgG (eB144, eBiosience, 18‐8817‐33) coupled to horseradish peroxidase in a dilution of 1:1,000. In between blocking and incubation steps, plates were washed 3 time with washing buffer (PBS + 0.05% Tween 20) and sera and secondary antibodies were diluted in this buffer. Plates were developed with tetramethylbenzidin and the reaction was stopped by adding 0.5 M sulfuric acid. Absorbance was measured at 450 nm.

### Placenta histology

For each litter, two placentas from male and female offspring were collected at gd 18.5, fixed in 4% formaldehyde solution and embedded in paraffin. Placentas were then cut in 4 μm sections at the mid sagittal plate. To perform histomorphological evaluation of placental areas, placental tissue was processed using Masson‐Goldner trichrome staining. Placental labyrinth (L) and junctional zone (JZ) were quantified with the software ZEN v.3.3 (Jena, Germany) and placental ratio (L/JZ) was calculated. Quantification was conducted by two independent investigators.

### Statistics

Statistical parameters including biological replicates *n*, precision measures (mean ± SEM), and statistical significance are reported in the figures and figure legends. Data was tested for normal distribution and the hypothesis judged to be non‐zero with statistical significance when *P* < 0.05 by unpaired Students' *t*‐Test, Mann–Whitney‐*U* test, Ordinary one‐way ANOVA or Kruskal–Wallis test after testing for normal distribution. In figures, asterisks denote statistical significance (*P < 0.05; **P < 0.01; ***P < 0.001; ****P < 0.0001). Outliers were removed by using outlier removal function in GraphPad PRISM 9 (Dotmatics, Boston, MA) with a false discovery rate of 1%. Statistical analysis was performed in GraphPad PRISM 9.

## Author contributions

**Dennis Yüzen:** Conceptualization; data curation; formal analysis; validation; investigation; visualization; methodology; writing – original draft. **Christopher Urbschat:** Data curation; investigation. **Steven Schepanski:** Data curation; investigation; methodology. **Kristin Thiele:** Conceptualization; supervision; methodology; writing – review and editing. **Petra C Arck:** Conceptualization; funding acquisition; validation; writing – original draft; project administration; writing – review and editing. **Hans‐Willi Mittrücker:** Conceptualization; formal analysis; supervision; funding acquisition; validation; writing – original draft; writing – review and editing.

## Disclosure and competing interests statement

The authors declare that they have no conflict of interest.

## Supporting information



Expanded View Figures PDF

Source Data for Expanded View

PDF+

Source Data for Figure 1

Source Data for Figure 2

Source Data for Figure 3

Source Data for Figure 4

## Data Availability

No primary datasets have been generated and deposited.
