# Supplementary material for: Pregnancy‐induced transfer of pathogen‐specific T cells from mother to fetus in mice
Source: EMBO Rep. 2023 Aug 23;24(10):e56829. doi: 10.15252/embr.202356829 (PMC10561172; doi:10.15252/embr.202356829)
Supplement: Supplementary file 1 — Expanded View Figures PDF [file EMBR-24-e56829-s001.pdf]

Expanded View Figures

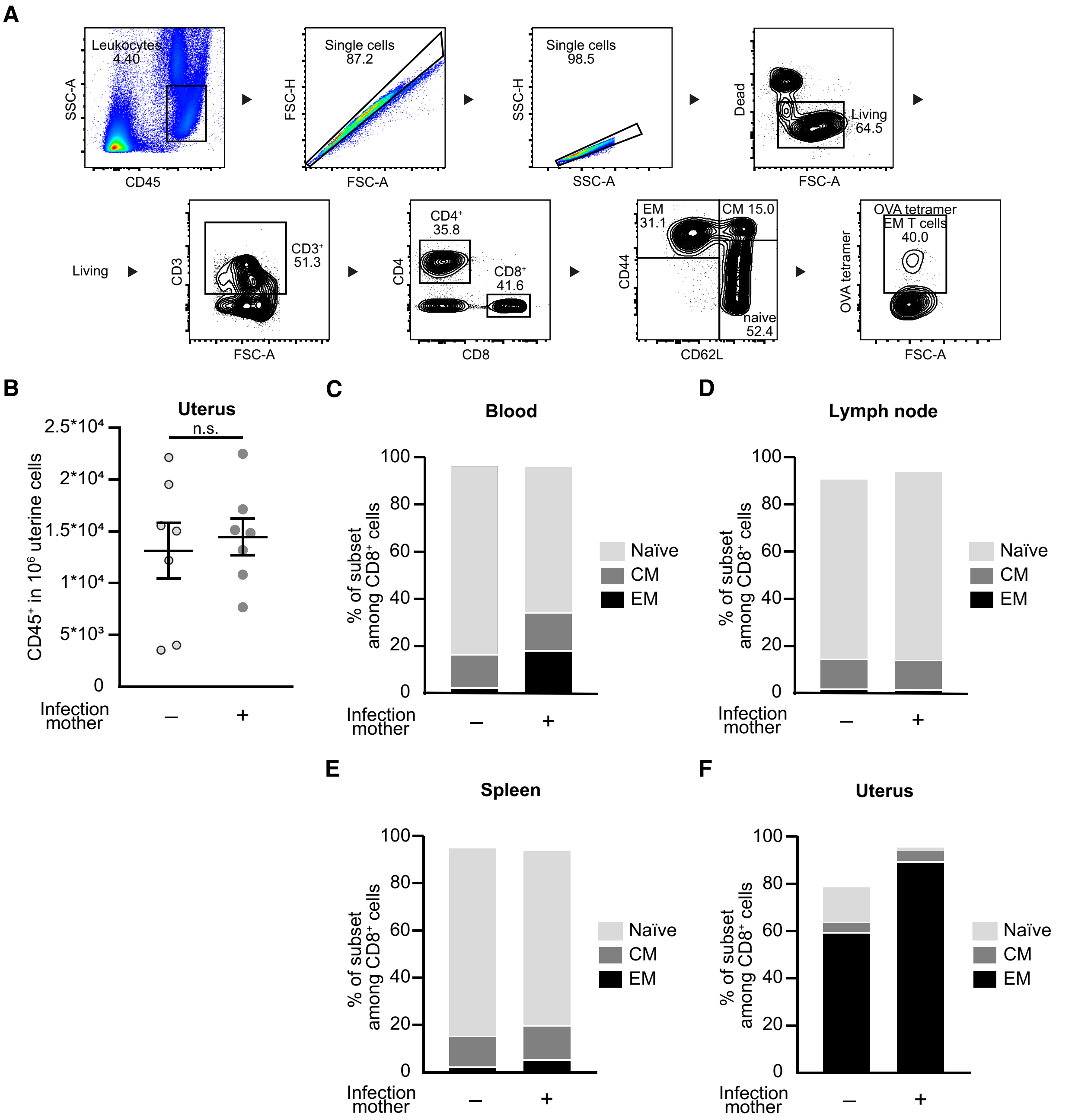

Figure EV1. Composition of CD8$^+$ T cell subpopulations after preconceptual infection.

A Gating strategy of CD8$^+$ T effector/effector memory cells. Blood of pregnant mice at gd 18.5 after preconceptual infection.

B Number of CD45$^+$ cells in 1 × 10^6 uterine cells on gd 18.5 after preconceptual infection of mothers in comparison with naïve mothers (n = 7 each, n: biological replicates).

C–F Percentage of CD8$^+$ T cell subpopulations: CD44$^+$CD62L$^-$ effector/effector memory (EM), CD44$^+$CD62L$^+$ central memory (CM), CD44$^-$CD62L$^+$ naïve; (C) peripheral blood (n = 6, n = 9), (D) uterus-draining lymph nodes (n = 6, n = 9), (E) spleen (n = 8, n = 7), (F) uterus (n = 7 each); n: biological replicates.

Data information: In (B), data are presented as mean ± SEM. In (C–F), data are presented as mean (B, D: Student's t-test; C, E, F: Mann–Whitney-U test).

© 2023 The Authors

Figure EV2. No differences in pregnancy outcome and MMc numbers in fetal organs after preconceptual infection of female mice.

A–D Pregnancy outcome parameter: (B) Percentage of fetal loss rate ($n = 8$, $n = 9$); (C) Number of implantations on gd 18.5 ($n = 7$, $n = 9$); (D) Male/female ratio of offspring ($n = 6$, $n = 7$); (E) Fetal weight on gd 18.5 ($n = 54$, $n = 55$); *n*: biological replicates.

E–G Placental evaluation: (E) Area of placental labyrinth (L) ($n = 12$ each); (F) Area of placental junctional zone (JZ) ($n = 12$–13); (G) Ratio of placental labyrinth to the junctional zone (L/JZ ratio) as an indicator for placental function on gd 18.5 ($n = 12$ each); *n*: biological replicates.

H Representative depiction of murine placenta assessment, highlighted for labyrinth (L, white) and labyrinth + junctional zone (black). Scale bar = 1,000 μm.

I Gating strategy of 'MMc-enriched' flow-through. Bone marrow of fetal mice at gd 18.5 born to a preconceptually infected mother.

J–L Numbers of MMc in 1×10^6 fetal cells on gd 18.5; (J) bone marrow ($n = 14$, $n = 13$), (K) spleen ($n = 14$, $n = 12$), (L) liver ($n = 13$, $n = 12$); *n*: biological replicates.

Data information: In (B–H), (J–L), data are presented as mean ± SEM. ****$P \leq 0.0001$ (C, E, G, K, L: Student's *t*-test; A, B, D, F, J: Mann–Whitney-*U* test).

 © 2023 The Authors

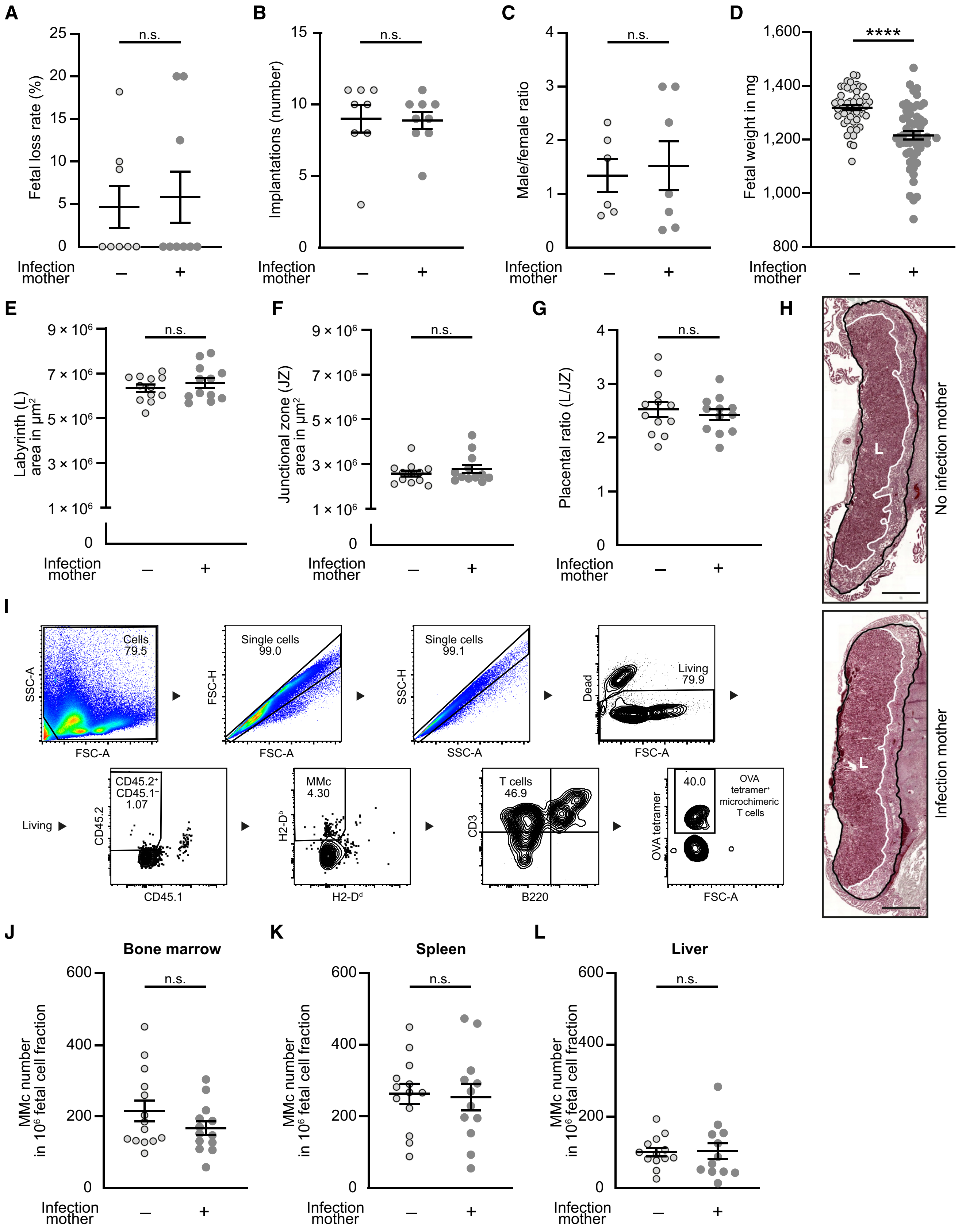
A
Fetal loss rate (%)
n.s.
B
Implantations (number)
n.s.
C
Male/female ratio
n.s.
D
Fetal weight in mg
****
Infection mother
E
Labyrinth (L) area in µm²
n.s.
F
Junctional zone (JZ) area in µm²
n.s.
G
Placental ratio (L/JZ)
n.s.
H
No infection mother
Infection mother
L
I
Cells 79.5
SSC-A
FSC-A
Single cells 99.0
FSC-H
Single cells 99.1
SSC-H
Dead
Living 79.9
Living
CD45.2⁺ CD45.1⁻ 1.07
CD45.2
CD45.1
MMc 4.30
H2-Dᵇ
H2-Dᵈ
T cells 46.9
CD3
B220
40.0
OVA tetramer⁺ microchimeric T cells
OVA tetramer
J
Bone marrow
K
Spleen
L
Liver
MMc number in 10⁶ fetal cell fraction
n.s.

Figure EV2.

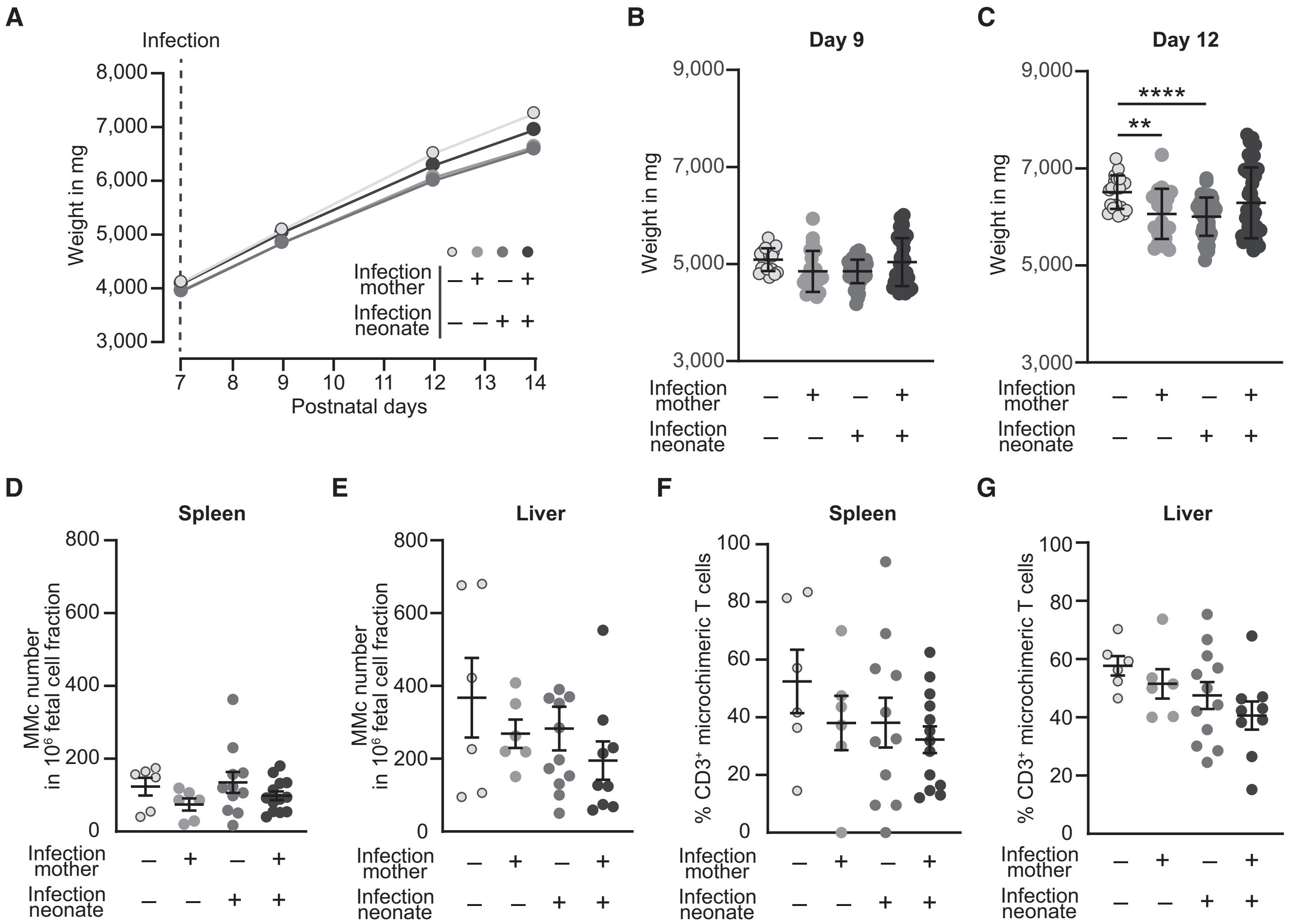

Figure EV3. Infection alters neonatal weight but does not influence MMc numbers.

A Body weight development after infection of neonates depicted as weight in mg.

B, C Neonatal body weight in mg (n = 20–55, n: biological replicates). (B) Day 9 after birth (2 days after infection), (C) day 12 after birth (5 days after infection).

D, E Number of MMc in 1×10^6 fetal cells on day 7 after infection; (D) spleen (n = 6–14), (E) liver (n = 6–11); n: biological replicates.

F, G Percentage of $CD3^+$ T cells among MMc; (F) spleen (n = 6–13), (G) liver (n = 6–12); n: biological replicates.

Data information: In (B–G), data are presented as mean ± SEM. $^{**}P \leq 0.01$; $^{****}P \leq 0.0001$ (B, F, G: Ordinary one-way ANOVA; C–E: Kruskal–Wallis test).
Source data are available online for this figure.

 © 2023 The Authors
